# Development and Characterization of Simple Sequence Repeat (SSR) Markers Based on RNA-Sequencing of *Medicago sativa* and *In silico* Mapping onto the *M. truncatula* Genome

**DOI:** 10.1371/journal.pone.0092029

**Published:** 2014-03-18

**Authors:** Zan Wang, Guohui Yu, Binbin Shi, Xuemin Wang, Haiping Qiang, Hongwen Gao

**Affiliations:** 1 Institute of Animal Science, Chinese Academy of Agricultural Sciences, Beijing, China; 2 Department of Grassland Science, College of Animal Science and Technology, Northwest A&F University, Yangling, Shaanxi, China; Shenzhen Institutes of Advanced Technology, China

## Abstract

Sufficient codominant genetic markers are needed for various genetic investigations in alfalfa since the species is an outcrossing autotetraploid. With the newly developed next generation sequencing technology, a large amount of transcribed sequences of alfalfa have been generated and are available for identifying SSR markers by data mining. A total of 54,278 alfalfa non-redundant unigenes were assembled through the Illumina HiSeqTM 2000 sequencing technology. Based on 3,903 unigene sequences, 4,493 SSRs were identified. Tri-nucleotide repeats (56.71%) were the most abundant motif class while AG/CT (21.7%), AGG/CCT (19.8%), AAC/GTT (10.3%), ATC/ATG (8.8%), and ACC/GGT (6.3%) were the subsequent top five nucleotide repeat motifs. Eight hundred and thirty- seven EST-SSR primer pairs were successfully designed. Of these, 527 (63%) primer pairs yielded clear and scored PCR products and 372 (70.6%) exhibited polymorphisms. High transferability was observed for ssp *falcata* at 99.2% (523) and 71.7% (378) in *M. truncatula*. In addition, 313 of 527 SSR marker sequences were *in silico* mapped onto the eight *M. truncatula* chromosomes. Thirty-six polymorphic SSR primer pairs were used in the genetic relatedness analysis of 30 Chinese alfalfa cultivated accessions generating a total of 199 scored alleles. The mean observed heterozygosity and polymorphic information content were 0.767 and 0.635, respectively. The codominant markers not only enriched the current resources of molecular markers in alfalfa, but also would facilitate targeted investigations in marker-trait association, QTL mapping, and genetic diversity analysis in alfalfa.

## Introduction

Alfalfa (*Medicago sativa* L.) is called the “Queen of Forages” and is the most important and widely planted forage legume for hay, pasture and silage in the world because of its high nutritional quality as well as its economic value. Cultivated alfalfa is a perennial autotetraploid (2n = 4x = 32), cross-pollinated and seed propagated crop [1]–[3]. Because of the tetrasomic inheritance, multiple allelelism and pronounced inbreeding depression of this species, improving forage quality and yield in this species is challenging. Clear distinction and identification between heterozygous and homozygous loci, such as these in alfalfa will rely on polymorphic codominant markers. The utilization of molecular breeding approaches especially marker-assisted selection will increase the efficiency of cultivar development to meet existing and future needs of alfalfa and forage crop production.

Microsatellites or simple sequence repeats (SSRs) are found throughout the eukaryotic genomes [4] and occur in both coding and noncoding regions [4]–[5]. SSRs have been one of the most useful molecular marker systems for their co-dominant inheritance, abundance in genomes, high reproducibility, and have been extensive used in molecular genetic research in alfalfa [6]–[8]. Compared with the development of genomic - SSRs which are identified from random genomic sequences, the development of EST-SSR identified from transcribed RNA sequences provides a fast, cost-effective, and reliable alternative strategy in the high through-put sequencing era [9]–[12]. Due to their existence in transcribed region of genome, EST-SSR markers can lead to the development of gene-based maps which may help to identify candidate functional genes and increase the efficiency of marker-assisted selection [13]–[14]. In addition, EST-SSRs show a higher level of transferability to closely related species than genomic SSR markers [15]. This higher level of cross-taxon marker would probably facilitate comparative genetic analyses. As a matter of fact, SSRs have been successfully developed from ESTs in many species [16]–[19].

SSR markers in genus *Medicago* germplasm were first reported by Diwan et al [20]. Since then, several studies have reported SSR markers which were used in alfalfa [8], [21]. But all of them were derived from the EST-SSRs [22]–[25], and bacterial artificial chromosome [26]–[27] in barrel medic (*M. truncatula*). He et al [21] developed 61 genomic SSR by constructing the genomic libraries of alfalfa. Wang et al developed 29 polymorphic SSR using 12,371 EST sequences from the National Centre for Biotechnology Information [28].

Here, we report the generation of a large expressed sequence dataset based on Illumina HiSeq™ 2000 sequencing data from the young leaves of alfalfa and the development of a large number of novel and efficient genic-SSR molecular markers. These developed SSR markers will provide a useful tool for marker-trait association, QTL mapping, and genetic diversity analysis in alfalfa.

## Materials and Methods

### Plant materials and DNA extraction

A total of 32 alfalfa cultivars / landraces, as well as a *falcata* accession and a barrel medic accession, were used in the study (Table S1). Six alfalfa cultivars/landraces were used to screen SSR markers developed from this study (Table S1). In addition, a *falcata* accession and a barrel medic accession were used to study the cross species transferability of SSR markers. Thirty alfalfa cultivars / landraces were used to assess the genetic diversity. Seeds were obtained from the National Herbage Germplasm Bank of China (Beijing) and Institute of Animal Science, Chinese Academy of Agricultural Science (Table S1).The leaf samples were collected by mixing equal amounts of leaf tissue from five plants of each accession. Genomic DNA from young leaves was extracted using the Hexadecyl trimethyl ammonium Bromide (CTAB) extraction method [29]. DNA quality was tested by 1% agarose gel electrophoresis. The working concentration of DNA was adjusted to 50 ng/μl.

### RNA isolation, library preparation, Illumina sequencing and *de novo* transcriptome assembly

*M. sativa* ssp *sativa*.cv. ‘Zhongmu No 1’ plants were used in the RNA-seq experiment. Plants were grown in pots in a greenhouse at 24°C with a 16 h light/8 h dark photoperiod cycle. After 6 weeks, samples of aboveground part were harvested and the excised shoots were immediately frozen in liquid nitrogen and stored at −80°C. Total RNA was extracted using a trizol reagent (Invitrogen, USA) following the manufacturer instructions. RNA samples were quantified and examined for protein contamination (Concentration, rRNA ratio (28 s/18 s) and RNA integrity) by Agilent 2100 bioanalyzer and protein contamination (A260/280 nm and A260/230 nm ratio) by a NanoPhotometer Pearl (Implen, Germany).

Poly (A) mRNA was isolated from the total sample RNA. Fragmentation buffer was added for interrupting mRNA to short fragments. Taking these short fragments as templates, random hexamer-primer was used to synthesize the first-strand cDNA samples. The second-strand cDNA was synthesized using buffer, dNTPs, RNaseH and DNA polymerase I. Short fragments were purified and resolved with Ethidium bromide (EB) buffer for end preparation and tailing A. After that, the short fragments were ligated to sequencing adapters. After the agarose gel electrophoresis, the suitable fragments were selected for the PCR amplification as templates. At last, the library was sequenced using Illumina HiSeq™ 2000. Raw reads were obtained after image data were transferred into sequenced reads by base calling. All low quality tags such as unknown nucleotides which was larger than 5% reads with adaptors were removed. The resulting high-quality sequences were assembled with the short reads assembling program-Trinityrnaseq (http://sourceforge.net/projects/trinityrnaseq). The paired-end raw reads were deposited into NCBI (Accession number: SRR944642).

### SSR loci identification

This unigene dataset obtained as described above was used for identification of potential SSRs using the MISA (http://www.pgrc.ipk-gatersleben.de/misa). In order to remove possible duplicates of published EST-SSRs, comparison was performed using the primers from the published EST-SSR markers [28] against the 4,926 unique SSR-containing sequences. Each set of sequences was compared by specialized NCBI blast program bl2seq using default parameters with the exception that the word size algorithmic parameter was changed from 28 to 16 due to the short length of the primers (18–28 bp). The criteria for identifying SSRs for all possible combinations of core sequences were 6, 5, 5, 4, and 4 repeats for di-, tri-, tetra-, penta-, and hexanucleotides, respectively.

### Primer design and PCR amplification

Primer pairs were designed using the Batch Primer 3 software (http://probes.pw.usda.gov/cgi -bin/batchprimer3/batchprimer3.cgi). The parameters for primers design were: (1) primer length from 18 to 28 with 23 as the optimum; (2) PCR product size from 150 to 300 bp; (3) annealing temperature from 55 to 65°C with an optimum of 60°C; (4) GC contents from 45% to 55%, with 50% as optimum. The primer pairs were synthesized by Sangon (Shanghai, China).

PCR amplifications of genomic DNA was carried out in a 25 μL reaction volume in a Thermal Cycler (BBI, Canada) containing 2.5 μL 10×PCR buffer (100 mM Tris-HCl pH 8.8 at 25°C; 500 mM KCl, 0.8% (v/v) Nonidet), 0.5 μL 10 mM dNTPs, 0.2 μL of Taq DNA polymerase (5 U/μL) (Sangon, Shanghai, China), 0.5 μl 10 μM of each primer, 2.0 μL 25 mM MgCl_2_, 1.0 μL of template DNA and 17.8 μL ddH_2_O. The following PCR profile was used: an initial denaturing for 8 min at 95°C, followed by 10 cycles of 95°C for 1 min, 60°C for 30 s, and 72°C for 45 s; and 20 cycles of 95°C for 45 s, 55°C for 30 s, and 72°C for 45 s; a final extension at 72°C for 6 min. PCR products for SSR primers screening were separated on 6% polyacrylamide denaturing gels. The gels were silver stained for SSR bands detection.

Multiplex PCR was designed and tested with products of different sizes and labeled with different fluorescent dyes. PCR reactions were carried out as described above. The following PCR profile was used: an initial denaturing for 8 min at 95°C, followed by 10 cycles of 95°C for 30 s, 60°C for 30 s, and 72°C for 30 s; and 20 cycles of 95°C for 30 s, 55°C for 30 s, and 72°C for 30 s; a final extension at 72°C for 6 min. PCR products of genetic diversity of 32 alfalfa cultivars were separated on an ABI3730xl DNA Analyzer (Applied Biosystems, Foster City, CA, USA). Fluorescence-labeled primers were synthesized at Applied Biosystems Company. Fragment sizes were determined using an internal size standard (LIZ500, ABI, USA), and the outputs were analyzed using GeneMapper software (http://www.appliedbiosystems.com.cn/).

Unigene sequences were initially aligned by Blastx to protein databases including nr, Swiss-Prot, KEGG and COG (e-value<0.00001), and then aligned by Blastn to nucleotide databases nt (e-value<0.00001), retrieving proteins with the highest sequence similarity with the given Unigenes along with their protein functional annotations.

### *In silico* mapping of alfalfa SSR markers

The SSR sequences were BLAST-searched against the genome sequences at Phytozome (http://www.phytozome.net/) to find each SSR marker's physical position, and the SSR markers were mapped according to their position on each barrel medic chromosome. The *in silico* map was drawn with Map Chart 2.0 [30].

### Statistical analyses

For the screening of EST-SSR markers, statistical analysis was carried out by treating the EST-SSRs in alfalfa as dominant markers. The data was scored as presence (1) or absence (0). Polymorphism information content (PIC) was calculated by PIC_CALC 0.6 (http://hi.baidu.com/luansheng1229/item/306815126d58e3a4feded5a4) according to Botstein et al [31].

For the phylogenetic analysis of 30 Chinese alfalfa cultivated accessions using 36 randomly selected polymorphic EST-SSRs primers. Individual tetraploid genotypes were scored from microsatellite banding patterns in the electropherograms following the Microsatellite DNA Allele Counting-Peak Ratios (MAC-PR) method of Esselink et al [32]. Allele frequencies, Allelic richness (A, the number of alleles at a locus), and observed heterozygosities (H_O_) were calculated using AUTOTET [33]. As it is unknown if double reduction occured in alfalfa, expected heterozygosity was calculated considering both random mating with random chromosomal segregation (He(Ce)) and random mating and some level of chromatid segregation (i.e. the default value of maximum double reduction where a = 1/7) [33], He(Cd). PIC was calculated by PIC_CALC 0.6. Pairwise genetic distances [34] between 30 cultivars were computed and created a neighbor-joining dendrogram, using the software program POPULATIONS v. 1.2.31 (http://bioinformatics.org/~tryphon/populations/). The resulting dendrogram was drawn by Treeview [35].

## Results and Discussion

### Frequency and distribution of EST-SSRs in alfalfa

Using high-throughput RNA-seq technology, more than 56.92 million paired-end raw reads were obtained (Accession number: SRR944642). After removal of the low quality reads, 53.85 million clean reads remained. With the aid of short reads assembling program-Trinity, these clean reads were de novo assembled into 122,127 contigs, further into, 55,881 unigenes, ranging from 200 to 3,000 bp with a mean length of 630 bp. The total length of the unigenes was over 3.52 G bp.

A total of 1,603 ESTs highly similar to those published by NCBI were removed and finally 54,278 unigenes with an average length of 633 bp were used for further SSR mining. This represented approximately 3.4%–4.3% (34.38 Mb) of the estimated 800–1000 Mb alfalfa genome [36]. Of the 54,278 unigenes, 3,903 (7.2%) were identified as containing SSRs (Table 1). This frequency was consistent to the frequency range of 2.65 to 10.62% which has been reported in 49 dicot species [37]. Frequency of EST-SSRs in various plant genomes is significantly influenced by the repeat length and the criteria used for mining the SSRs in the database [38]. The present study indicated a relatively higher abundance of EST-SSRs as compared to earlier reports in barrel medic (3.0%) (24) and alfalfa (6.1%) [28]. Of the 3,903 SSR-containing ESTs, 488 (12.5%) contained two or more SSRs, and 285 (7.3%) comprised compound SSR repeats (Table 1). The frequency and distribution of EST-SSRs in alfalfa are shown in Table S2. A total of 4,993 EST-SSR markers were identified from the 3,903 SSR-containing ESTs. On an overall average, one SSR was identified per 7.47 kb. This was quite comparable to the frequency reported in *Coffea* (7.73 kb) [38], peanut (*Arachis hypogaea*) (7.3 kb) [39], sweet potato (*Ipomoea batatas*) (7.1 kb) [40], cassava(*Manihot esculenta*) (7.0 kb) [41].While a lower frequency was found in barrel medic (1.8 kb) [42], rubber tree (*Hevea brasiliensis*) (2.25 kb) [18], pepper (*Capsicum annuum*)(3.8 kb) [43], and in tea(*Camellia sinensis*) (3.5 kb) [44]. However, a direct comparison of abundance estimation and frequency occurrence of SSRs in different reports is difficult due to the fact that the estimates were dependent on the SSR search criteria, the size of the dataset, the database mining tools and the EST sequence redundancy [14].

**Table 1:** Summary of EST-SSR found in alfalfa.

Searching items	Numbers
Total number of sequences examined	54,278
Total size of examined sequences (bp)	34,375,141
Total number of identified SSRs	4,493
Number of sequences containing SSRs	3,903
Number of sequences containing more than 1 SSR	488
Number of SSRs present in compound formation	285
Di-nucleotide	1,315
Tri- nucleotide	2,548
Tetra- nucleotide	161
Penta- nucleotide	295
Hexa- nucleotide	174

Generally, the proportion of different SSR unit sizes was not evenly distributed. In the present study, of the 4,493 EST-SSRs, tri-nucleotide repeats (56.71%) was the most abundant motif and followed by di-nucleotide (29.27%), penta- nucleotide (6.57%), hexa-nucleotide (3.87%), and tetranucleotide (3.58%), respectively (Table 2). There was a large proportion of both di- and trinucleotides (86%) while the rest amounted to less than 14%. This is consistent with the EST-SSRs distributions reported in alfalfa [28], barrel medic [42], chickpea (*Cicer arietinum*) [17], castor bean(*Ricinus communis*) [19], peanut [39], sweet potato [40], and date palm(*Phoenix dactylifera* L.) [45]. It was proposed that the deleterious effects of the frame-shift mutation in translated regions could suppress the expansion or contraction of di-nucleotide repeat length in exons [46]. This might help to explain the dominant motifs of tri-nucleotide found in this study.

**Table 2:** Summary frequencies of different SSR repeat motif types related to variation of repeat unit numbers in alfalfa EST-SSR loci.

Repeat number	Di-	Tri-	Tetra-	Penta-	Hexa-	Total	%
4				266	165	431	9.59
5		1411	128	26	5	1,570	34.94
6	418	732	30	2	1	1,183	26.33
7	290	356	2		1	649	14.44
8	175	39	1		2	217	4.83
9	190	3				193	4.30
10	160			1		161	3.58
11	78	1				79	1.76
12	3	2				5	0.11
13						0	0.00
14						0	0.00
15		1				1	0.02
16		2				2	0.04
17						0	0.00
18						0	0.00
19						0	0.00
20						0	0.00
≥21	1	1				2	0.04
Total	1,315	2,548	161	295	174	4,493	
%	29.27	56.71	3.58	6.57	3.87		

Based on the length of each SSR sequence, the microsatellites have been grouped into two classes: class I (12≤ n<20 nucleotides) and class II (n≥20 nucleotides) [47].The longer the SSR repeats were, the higher the rate of polymorphism in transcripts was observed. This observation was correlated to higher mutation rates because of more frequent slipped strand mispairing between the chromosomes [48]. Based on the Table 2, most SSR repeats had sequence length in class I, accounting for 71.6% (3,216 SSRs) of total SSRs, while 1,277 SSRs (28.4%) had longer sequences of repeats in class II.

A total of 171 SSR motifs were identified. The di-, tri-, tetra-, penta- and hexanucleotide repeats had 4, 10, 20, 52 and 85 types, respectively (Table S2). The most abundant type was AG/CT (21.7%), followed by AAG/CTT (19.8%), AAC/GTT (10.3%), ATC/ATG (8.8%), ACC/GGT (6.3%), and AC/GT (5.6%) (Table S2). AG/CT and AAG/CTT were the most frequent di- and trinucleotide motif types, respectively. Similar results have been reported in many dicotyledonous species [17]–[19], [22], [41], [49].The remaining motifs represented a frequency of 27.5%. Generally, CG/GC and CCG/CGG are very rare in dicotyledonous plants but common in monocots [40]. In the present study, there were 3 CG/GC and 21 CCG/CGG repeats out of the 4,993 total SSRs. This was also consistent to the previous reports on barrel medic [42] and alfalfa [28] which indicated no or rare CG/GC, and CCG/CGG repeats.

### Development, validation and transferability of EST-SSR markers

A total of 837 primer pairs were designed from 3,903 SSR-containing ESTs. The remaining ESTs did not qualify for primer design due to either too short flanking sequences of the SSRs (generally <40 nucleotides) or inability to fulfill the criteria for primer design. The 837 EST-SSR primer pairs including 99 di-, 649 tri-, 13 tetra-, 36 penta- and 40 hexa-nucleotide repeats were assayed in six ssp. sativa accessions through denaturing PAGE (Polyacrylamide gel electrophoresis) (Table 3). In total, 527 primer pairs (63%) produced clear amplicons of expected size, covering 66 di-, 414 tri-, 8 tetra-, 19 penta- and 20 hexa-nucleotide repeats (Table 3). These results were similar to the success rate of 60-90% amplification in previous studies [15], [39]–[40]. A total of 1,703 alleles were detected with an average of 3.2 alleles per SSR marker (Table S3). The remaining EST-SSR primer pairs that either had no amplification or produced a number of faint bands indicative of non-specific amplifications or gave larger and smaller amplicons than expected sizes were excluded from further analysis. The amplification failure might have been due to the presence of an intron within the primer sequences that prevented primer annealing or a large intron in the flanking region that disrupted PCR extension.

**Table 3:** The evaluation of microsatellite primer pairs for different repeat classes.

Class	Tested primers (%)	Scorable primers (%)	Polymorphic primer (%)	Mean of alleles per polymorphic locus ± standard derivation	PIC ± standard derivation
Di-	99 (11.8)	66 (12.5)	56 (84.8)	4.7±1.64	0.640±0.122
Tri-	649 (77.5)	414 (78.6)	274(66.2)	3.8±1.48	0.570±0.156
Tetra-	13 (1.6)	8 (1.5)	8(100.0)	4.4±0.92	0.670±0.087
Penta-	36 (4.3)	19 (3.6)	16(84.2)	3.9±1.20	0.607±0.114
Hexa-	40 (4.8)	20 (3.8)	18(90.0)	4.3±1.36	0.645±0.106
Total or average	837	527	372 (70.6)	3.99	0.588

To determine the possible functions of the 527 EST-SSRs, they were subjected to BLAST analysis. The results showed that 54.1% showed homology to a large number of known proteins. While the remaining EST-SSRs resulted in no hit (7%) or hypothetical protein (38.9%) which may represent specific transcripts for alfalfa, which is yet to be characterized for their putative functions. The functional annotations of these EST-SSRs are listed in Table S3.

Among the 527 primer pairs, 372 (70.6%) polymorphic EST-SSR markers were identified containing 56 di-, 274 tri-, 8 tetra-, 16 penta- and 18 hexa motifs while the other 155 primer pairs were monomorphic (Table 3). Compared to other plants, the polymorphic ratio of EST-SSR markers in alfalfa was higher than that in castor bean (41%) [19], sweet potato (41.9%) [40]. These polymorphic EST-SSRs generated a total of 1,483 alleles with an average of 4.7, 3.8, 4.4, 3.9, and 4.3, respectively for 261 di-, 1047 tri-, 35 tetra-, 63 penta- and 77 hexanucleotide SSR markers (Table 3). The number of alleles generated across the SSR loci ranged from 2 to 10 alleles while most of them had 2 to 5 alleles with a mean of 4 alleles per locus. This result was similar to a previous report in alfalfa [21]. Across 372 polymorphic loci, PIC value ranged from 0.215 to 0.847 with an average of 0.588 suggesting that the developed EST-SSRs were highly polymorphic (Table S3).

SSR markers with dinucleotide repeats were more polymorphic than trinucleotide repeats in several plant species [50]–[51]. As reported previously, di-, tetra- and penta- repeat loci mainly occurred within UTR regions, while tri- and hexarepeat loci SSRs mainly occurred within exon regions [19]. Unsurprisingly, di-, tetra-, and penta-motif loci presented higher polymorphic rates than tri-, and hexarepeat motif loci in alfalfa.

Because of the conserved nature of EST-SSRs, relatively low polymorphism was detected as compared with SSRs derived from genomic libraries [52]–[53]. He et al [21] reported 78.2% polymorphic genomic SSR primers (44, and 17 for di- and trinucleotide SSRs, respectively). In the present study, 70.6% SSR loci showed slightly lower polymorphism compared to the genomic SSR primers (Table 3) [21]. If only di- and trinucleotide SSRs were considered, their polymorphism rate was 68.8%. Similar results were also found for the mean number of alleles per polymorphic locus. On average, di- and tri-nucleotide genomic SSRs generated 5.2 and 4.8 alleles per primer pair, respectively, which is higher than 4.7 and 3.8 alleles per primer pair for EST-SSRs (Table 3) [21].

To validate the cross-species transferability, 527 alfalfa EST-SSRs markers were also used to amplify two accessions from ssp. *falcata* and barrel medic. The transferability rates of these EST-SSRs were 99.2% (523) in ssp. *falcata* and 71.7% (378) in barrel medic (Table S3).

### *In silico* mapping of SSR loci

*In silico* mapping is convenient, saves labor, and is low cost in contrast to the classical population-based mapping whenever a genome sequence is available. Of the 527 amplified alfalfa SSR markers, 313 were located on the eight barrel medic chromosomes, while 4 showed no homology in the barrel medic genome sequences, and 210 just hit the sequence but were not located on the barrel medic chromosomes. The number of markers on chromosomes 1–8 were 35, 32, 35, 45, 59, 16, 52, and 39, respectively (Figure S1). The in silico mapping used here succeeded in mapping 59.4% (313/527) of alfalfa SSR markers on the barrel medic chromosomes; a relatively low ratio compared with the 96.3% obtained by Li et al [54] in sorghum.

### Phylogenetic analysis of 30 Chinese alfalfa cultivated accessions

The 36 randomly selected EST-SSRs primer pairs from 372 polymorphic SSRs were used to assess the phylogenetic analysis of 30 Chinese alfalfa cultivated accessions. A total of 199 scored alleles were generated. Thirteen of 36 (36%) primer pairs amplified four alleles each with an average of 5.4 alleles per primer pair while the most polymorphic primer pair (MSE470) generated ten alleles (Table 4). The observed heterozygosity (Ho) ranged from 0.344 (MSE653) to 0.96 (MSE590) with a mean of 0.767 (Table 4). The mean expected heterozygosity He (Ce) and He (Cd) were 0.679 and 0.634, respectively. The PIC values at each locus varied from 0.363 for locus MSE437 to 0.812 for MSE470 with a mean of 0.635 (Table 4).

**Table 4:** Characterization of 30 alfalfa cultivars by 36 EST-SSR markers.

Locus	A^a^	Size range(bp)	Ho	He(Ce)^b^	He(Cd)	PIC
MSE3	4	170–182	0.828	0.718	0.67	0.667
MSE8	4	183–195	0.444	0.412	0.384	0.382
MSE18	5	145–157	0.922	0.774	0.722	0.736
MSE83	6	182–199	0.85	0.813	0.759	0.786
MSE99	8	150–177	0.783	0.66	0.616	0.609
MSE112	4	164–185	0.95	0.746	0.696	0.698
MSE114	4	170–188	0.739	0.62	0.578	0.554
MSE115	5	167–183	0.867	0.747	0.698	0.710
MSE116	4	176–188	0.738	0.709	0.662	0.660
MSE117	9	180–198	0.845	0.785	0.733	0.756
MSE137	5	171–183	0.5	0.548	0.512	0.514
MSE145	6	177–198	0.73	0.676	0.63	0.625
MSE157	7	160–172	0.856	0.768	0.717	0.731
MSE160	7	187–211	0.917	0.794	0.741	0.762
MSE191	5	149–168	0.793	0.664	0.62	0.610
MSE203	5	204–223	0.889	0.728	0.68	0.680
MSE204	4	146–167	0.761	0.639	0.597	0.572
MSE231	6	187–205	0.822	0.714	0.666	0.678
MSE437	4	169–180	0.372	0.396	0.37	0.363
MSE470	10	173–197	0.894	0.832	0.776	0.812
MSE477	4	142–154	0.844	0.706	0.659	0.651
MSE479	6	157–175	0.933	0.764	0.713	0.726
MSE482	4	168–177	0.689	0.603	0.563	0.551
MSE493	4	185–197	0.792	0.688	0.642	0.638
MSE501	4	145–160	0.943	0.748	0.698	0.701
MSE503	4	194–204	0.778	0.7	0.654	0.648
MSE546	8	174–195	0.672	0.638	0.595	0.596
MSE562	7	149–167	0.937	0.768	0.717	0.731
MSE568	7	172–199	0.75	0.684	0.639	0.642
MSE573	4	183–195	0.617	0.574	0.535	0.524
MSE586	5	183–203	0.8	0.72	0.672	0.673
MSE590	5	189–204	0.96	0.786	0.734	0.752
MSE609	5	159–171	0.789	0.671	0.626	0.616
MSE622	4	171–186	0.428	0.431	0.403	0.398
MSE629	7	172–193	0.833	0.738	0.689	0.702
MSE635	4	174–192	0.722	0.63	0.588	0.583
MSE653	4	193–202	0.344	0.523	0.488	0.455
MSE775	6	128–172	0.806	0.698	0.651	0.650

Note: A (Allelic richness), Ho (Observed heterozygosity), He (Expected heterozygosity), PIC (Polymorphism information content.

b expected heterozygosity assuming random mating and random chromosomal segregation (He), assuming random mating and non random chromatid segregation (He(ce)), i.e..

a double reduction frequency of a = 1/7 (Wrike and Weber 1986) (He(cd)).

Genetic relationships between the cultivars were assessed by constructing a neighbor-joining dendrogram using Nei's genetic distance data. The dendrogram grouped the 30 accessions into three main clusters (Figure S2). Fifteen accessions, including 11 ssp *sativa* cultivar/landraces (“Aohan”, “Baoding”, “Gongnong No 1”, “Longdong”, “Tianshui”, “Tumu No 2”, “Zhaodong”, “Zhonglan No 1”, “ Zhongmu No 1”, “Zhongmu No 2”, “Gannong No 5”), three nssp *varia* cultivars (“Gannong No 1”, “Gannong No 2”, and “Xinmu No 1”), and “Longmu No 801”, formed the first cluster. The cluster II comprised six ssp *sativa* cultivars (“Beijiang”, “Gannong No 3”, “Huaiyin”, “Longzhong”, “Neimeng Zhungeer”, “Yuxian”) and landraces, one nssp *varia* cultivars “Caoyuan No 3”. The cluster III contained six ssp *sativa* cultivars (“Gannong No 4”, “Gongnong No 2”, “Jinnan, “Pianguan”, “Xinjiang Daye”, “Xinmu No 2”) and landraces, two nssp *varia* cultivars (“Caoyuan No 2”, and “Tumu No 1”). The results showed that six nssp *varia* cultivars were not grouped together but dispersed into three clusters with other ssp *sativa* cultivars/landraces. The clustering results represented the genetic relatedness among the 30 accessions. The Chinese alfalfa cultivars genotyped in the experiment are synthetic populations developed through either phenotypic recurrent selection or natural mass selection with many parents which are adapted to local environments and exhibited increased productivity or/and resistance to specific abiotic or biotic stresses. The recurrent selection methods involving multiple hybridizations and selection activities with available germplasm in nssp *varia* and ssp *sativa* in breeding programs explains well why nssp *varia* cultivars and ssp *sativa* cultivars do not form separate clusters.

## Conclusions

In this study, 527 EST-SSR markers yielded clear and scorable PCR products and 372 exhibited polymorphisms for alfalfa through RNA-seq and 313 of 527 EST-SSR markers were mapped onto the eight *M. truncatula* chromosomes by *in silico* mapping. The alfalfa EST-SSR markers showed a high level of polymorphism and were highly transferable in related species. These EST-SSR markers will facilitate genetic and genomic investigations in marker-trait association, QTL mapping, and genetic diversity analysis.

## Supporting Information

Figure S1 ***In silico*** **mapping of 313 alfalfa SSR markers to** ***M. truncatula*** **chromosome.**
(TIF)

Figure S2 **The Neighbor-joining tree of the 30 alfalfa cultivars based on 36 EST-SSR markers.** The dendrogram shows the genetic relationships among 30 Chinese alfalfa cultivated materials.
(TIF)

Table S1 **Germplasm accessions used in this study of alfalfa.**
(XLSX)

Table S2 **Frequencies of different repeat motifs in EST-SSRs from alfalfa.**
(XLSX)

Table S3 **Characteristics of alfalfa EST-SSR primers used in this study.**
(XLSX)
