# Correction: Development and Characterization of Simple Sequence Repeat (SSR) Markers Based on RNA-Sequencing of *Medicago sativa* and *In silico* Mapping onto the *M. truncatula* Genome

**DOI:** 10.1371/journal.pone.0103682

**Published:** 2014-07-21

**Authors:** The PLOS ONE Staff

In the Funding section, the grant number from the funder Agricultural Science and Technology Innovation Program is listed incorrectly. The correct grant number is: ASTIP-IAS10.
