# Supplementary figures and images for: Development and Characterization of Simple Sequence Repeat (SSR) Markers Based on RNA-Sequencing of Medicago sativa and In silico Mapping onto the M. truncatula Genome
Source: PLoS One. 2014 Mar 18;9(3):e92029. doi: 10.1371/journal.pone.0092029 (PMC3958398; doi:10.1371/journal.pone.0092029)

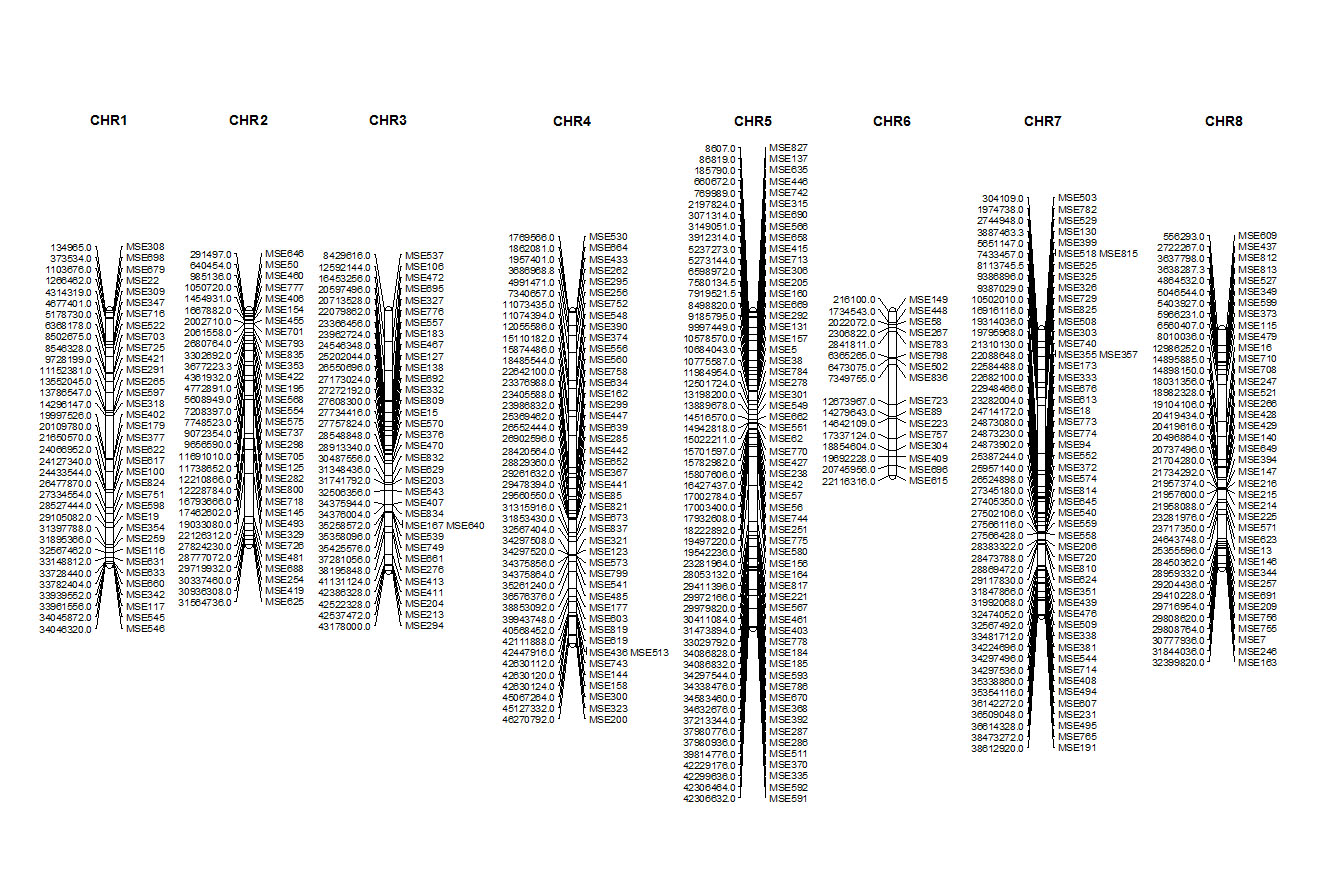

Supplement: Figure S1 — In silico mapping of 313 alfalfa SSR markers to M. truncatula chromosome. (TIF) [file pone.0092029.s001.tif]

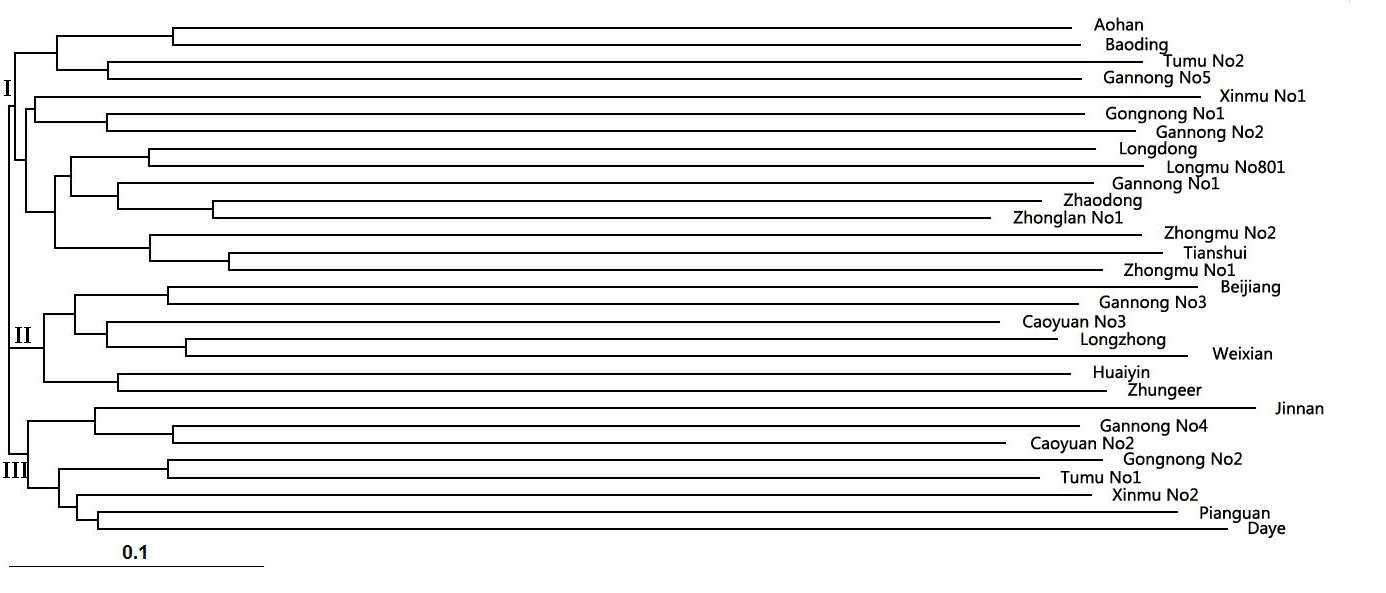

Supplement: Figure S2 — The Neighbor-joining tree of the 30 alfalfa cultivars based on 36 EST-SSR markers. The dendrogram shows the genetic relationships among 30 Chinese alfalfa cultivated materials. (TIF) [file pone.0092029.s002.tif]
